# Final results of the PräVAC trial: prevention of wound complications following inguinal lymph node dissection in patients with penile cancer using epidermal vacuum-assisted wound closure

**DOI:** 10.1007/s00345-020-03221-z

**Published:** 2020-05-05

**Authors:** Sebastian C. Schmid, Anna K. Seitz, Bernhard Haller, Hans-Martin Fritsche, Toni Huber, Maximilian Burger, Jürgen E. Gschwend, Tobias Maurer

**Affiliations:** 1 Department of Urology, Rechts der Isar Medical Center, Technical University of Munich, Ismaningerstr. 22, 81375 Munich, Germany; 2 Department of Urology, Universitätsklinikum Würzburg, Würzburg, Germany; 3 Institute of Medical Informatics, Statistics and Epidemiology, Technical University of Munich, Munich, Germany; 4 Department of Urology, Universitätsklinikum Regensburg, Regensburg, Germany; 5 Department of Urology and Martini-Klinik, University Medical Center Hamburg-Eppendorf, Hamburg, Germany

**Keywords:** Inguinal surgery, Penile cancer, Lymphorrhea, PraeVac, Closed incision negative pressure

## Abstract

**Purpose:**

Inguinal lymphadenectomy in penile cancer is associated with a high rate of wound complications. The aim of this trial was to prospectively analyze the effect of an epidermal vacuum wound dressing on lymphorrhea, complications and reintervention in patients with inguinal lymphadenectomy for penile cancer.

**Patients and methods:**

Prospective, multicenter, randomized, investigator-initiated study in two German university hospitals (2013–2017). Thirty-one patients with penile cancer and indication for bilateral inguinal lymph node dissection were included and randomized to conventional wound care on one side (CONV) versus epidermal vacuum wound dressing (VAC) on the other side.

**Results:**

A smaller cumulative drainage fluid volume until day 14 (CDF) compared to contralateral side was observed in 15 patients (CONV) vs. 16 patients (VAC), with a median CDF 230 ml (CONV) vs. 415 ml (VAC) and a median maximum daily fluid volume (MDFV) of 80 ml (CONV) vs. 110 ml (VAC). Median time of indwelling drainage: 7 days (CONV) vs. 8 days (VAC). All grade surgery-related complications were seen in 74% patients (CONV) vs. 74% patients (VAC); grade 3 complications in 3 patients (CONV) vs. 6 patients (VAC). Prolonged hospital stay occurred in 32% patients (CONV) vs. 48% patients (VAC); median hospital stay was 11.5 days. Reintervention due to complications occurred in 45% patients (CONV) vs. 42% patients (VAC).

**Conclusions:**

In this prospective, randomized trial we could not observe a significant difference between epidermal vacuum treatment and conventional wound care.

## Introduction

Penile cancer is a rare disease with an incidence of 1/100.000/year in Europe, with an age peak around the sixth decade. Invasive penile cancer is an aggressive disease with a high risk of metastasis, with local treatment adapted to tumor extension [1]. The primary landing site for metastasis are inguinal lymph nodes. Even in the case of clinically normal inguinal lymph nodes, the risk for micrometastasis is around 25% in patients with intermediate or high-risk penile cancer (≥ pT1 G2). In these patients, a surgical lymph node staging is recommended using inguinal lymphadenectomy [2]. It can be performed either as dynamic sentinel node biopsy or as modified (limited) inguinal lymphadenectomy. In patients with clinically suspicious (palpable or visible) inguinal lymph nodes, a radical inguinal lymphadenectomy is advised [3]. Although inguinal lymphadenectomy is a life-saving procedure, there is a relevant fraction of patients that does not undergo this procedure. Even in specialized centers, 25% of patients are not treated according to guideline recommendations leading to a significantly higher risk of death compared to guideline-adherent treatment [4, 5]. While the reasons for avoiding inguinal lymphadenectomy are unclear, the associated high morbidity is likely to contribute to this situation. Common wound problems include prolonged lymph secretion, lymphocele formation and wound dehiscence with secondary healing. These problems are observed in 25–70% of patients [6–11]. There have been different attempts to reduce the morbidity of this procedure. With regard to surgical technique, ligation is preferred over diathermic coagulation [12]. Another approach to reduce surgical trauma is dynamic sentinel node biopsy during or after primary surgery [13] to selectively identify the first draining nodes of the primary tumor [14]. For this method, a high negative predictive value of 99% and a sensitivity of 88% in regard to inguinal metastasis were reported in a recent metaanalysis [15].

Another method to prevent wound problems is the use of subatmospheric pressure or vacuum therapy in the form of an epidermal vacuum wound dressing. Epidermal vacuum therapy is applied on the closed wound and generates negative pressure in the wound thus stabilizing the wound and inducing healing processes. In 2012, our group presented the results of a retrospective analysis of 24 patients treated with epidermal vacuum wound dressing or conventional wound care after inguinal lymphadenectomy due to penile cancer [16]. This retrospective analysis showed promising results, with epidermal vacuum treatment (VAC) resulting in significantly fewer complications such as the formation of lymphoceles (62% vs. 20%), persistent lymphorrhea (45% vs. 7%) or lymphedema of the lower extremity (46% vs. 0%) (*p* = 0.032). In the retrospective analysis, reinterventions had to be performed in 23% of inguinal wounds (four patients) treated with conventional wound care and for 7% of inguinal wounds (one patient) treated with epidermal VAC (*p* = 0.631). To confirm this retrospective data, we conducted a prospective, randomized, multicenter trial of epidermal vacuum wound dressing (VAC) versus conventional wound care (CONV) following inguinal lymph node dissection to prospectively analyze the effect on lymphorrhea, complications and reinterventions in patients with penile cancer. The rationale for choosing epidermal vacuum treatment was the hypothesis that sub-atmospheric pressure might lead to compression and closure of lymphatic vessels as well as stabilization of the wound and removal of wound fluid, thereby reducing wound complications.

## Patients and methods

The study was planned as a prospective, randomized, multicenter, investigator-initiated trial in Germany. The trial was registered in the German clinical trials register DRKS (https://www.drks.de; DRKS00005257) and approved by the relevant legal authority (EUDAMED CIV-12-07-008204). The trial was approved by the ethics committee of the faculty of medicine (5543/12), Technical University Munich, Munich, Germany. The trial was performed in accordance with the Declaration of Helsinki. Patients were included after information and signing of the informed consent form. For each participating patient, intervention and control side were randomly allocated based on a computer-generated randomization list with a block size of ten. Planned accrual was 100 patients, with preplanned interim analyses after 25 and 50 patients. Participating centers included the Department of Urology, Rechts der Isar Medical Center, Technical University of Munich, Munich, Germany and Department of Urology, Universitätsklinikum Regensburg, Regensburg, Germany.

Aim of the study was to prospectively analyze the effect of an epidermal vacuum wound dressing on lymphorrhea, complications and reinterventions in patients with inguinal lymphadenectomy for penile cancer. Inclusion/exclusion criteria and endpoints are shown in Table 1.

**Table 1:** Table of inclusion/exclusion criteria and trial endpoints

Inlcusion criteria	Exclusion criteria
Patients with penile cancer	Status post inguinal surgery (e.g. vascular surgery) or any medical conditions leading to an impaired inguinal lymph drainage. Status post repair of inguinal hernia was no exclusion criterion, if date of surgery was > 3 months in the past and no swelling or edema was detectable
Indication for inguinal lymphadenectomy according to EAU guideline: tumor stage ≥ pT1 G2 or palpable inguinal lymph nodes	Allergy to acrylic adhesive
	Patients who are not able to give informed consent
	Age < 18 years
Primary endpoint	
Cumulative drainage fluid volume (CDF) in milliliter (ml) until removal of the drainage (at the latest up to day 14 post surgery)	
Secondary endpoints	
Maximum of daily fluid volume (MDFV)	
Days until removal of drainage	
Length of hospital stay	
Wound-associated complications during study treatment	
Need for reintervention due to wound complications within the first 90 postoperative days	
Postoperative pain and patient satisfaction (determined by visual analogue scale)	

### Study treatment

**Fig. 1:**
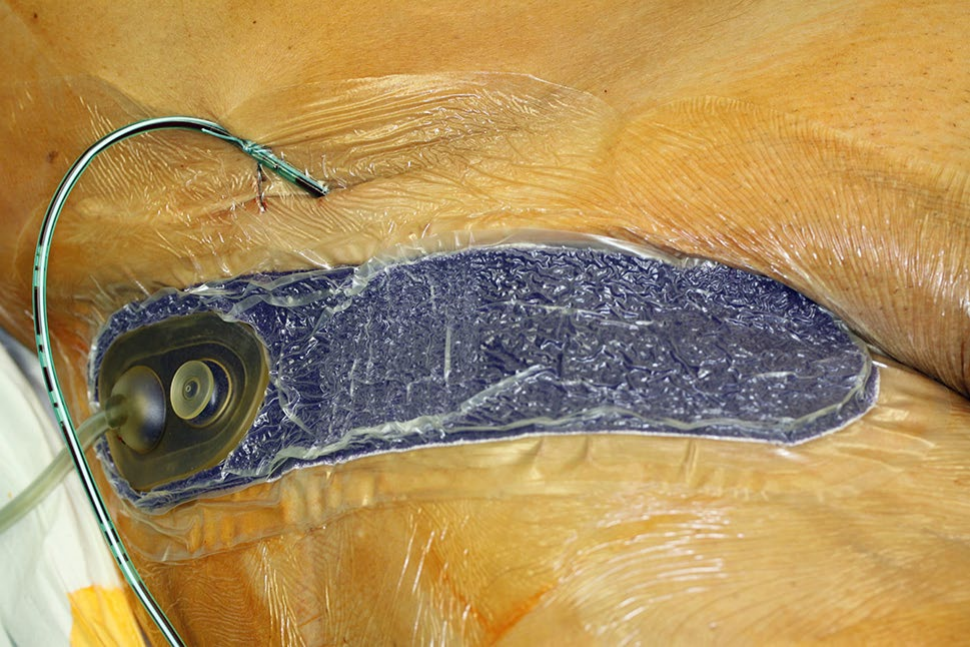
Epidermal vacuum treatment of the groin with the “PREVENA™ Incision Dressing” system, which is applied on the closed wound

Included penile cancer patients were treated by inguinal lymphadenectomy by experienced surgeons only in accordance with the EAU guideline [3] either as (modified) bilateral inguinal lymphadenectomy or as bilateral dynamic sentinel node biopsy. Subsequently, postoperative wound dressings were applied. Conventional wound care (CONV) treatment consisted of a subcutaneous suction drain as well as pressure dressings over the stapled closed wound while the epidermal vacuum (VAC) treatment consisted of a subcutaneous suction drain and a stapled closed wound and an epidermal vacuum wound dressing (PREVENA™ Incision Dressing, KCI, an Acelity company, San Antonio, Texas, USA) over the stapled closed wound (Fig. 1). To analyze the effect of epidermal vacuum therapy after bilateral inguinal lymphadenectomy and to reduce patient inherent confounders, both conventional wound treatment as well as epidermal vacuum treatment were applied within the same patient. The side of each treatment was determined by a computer-generated randomization list designed by a biostatistician prior to the start of the trial.

### Post-surgery assessment

After surgery, the epidermal vacuum dressing was removed after 7–8 days on the intervention side. On the control side, the pressure dressing was removed after 24 h. The suction drainage was removed if daily fluid volume was < 25 ml, but not earlier than three pod. Before discharge, bilateral inguinal sonography was performed. Wound associated complications were documented during the hospital stay. At time of discharge from hospital, patients were asked to score their maximum pain level on each side on a visual analogue scale from 0 to 10 (0—no pain; 10—maximum pain) and satisfaction level in regard to wound treatment on each side on a visual analogue scale from 0 to 10 (0—very unhappy; 10—very happy). The study treatment ended after discharge from the hospital. After 90 days, patients were contacted for follow-up using a structured questionnaire to account for wound associated complications and reinterventions.

### Statistical analysis

Primary aim of the study was to show, that—within a patient-intervention—VAC leads to a smaller cumulative drainage fluid volume of the respective side compared to CONV. It was planned to test, whether the probability for a smaller CDF observed on the side treated by VAC as compared to the side with CONV is larger than 70%. For the primary analysis, an exact binomial test comparing the observed proportion of patients with a smaller CDF by VAC to a probability of 70% was planned and performed. Two interim analyses were planned after 25 and 50 included patients with a two-sided significance level of α* = 1% and the final statistical test was intended to be performed on a significance level of *α* = 3%. A true probability for a lower CDF under VAC as compared to CONV within a patient of 85% was assumed, so 100 patients were planned to be included to reject the null hypothesis with a probability (= power) of 90%. Sample size calculation was performed using the software nQuery Advisor version 7.0.

For categorical data, absolute and relative frequencies are shown. Quantitative data are described by medians and interquartile ranges (IQR, 1st–3rd quartile) as most variables follow a skewed distribution. For comparisons of quantitative data, Wilcoxon-signed rank tests were performed, for binary data the exact McNemar test was conducted. To investigate associations between relevant patient characteristics and need for reintervention or occurrence of relevant complications (grade 3 or 4) on any side, Fisher’s exact test (for diabetes and smoking) or univariate binary logistic regression models (age, BMI) were used. A multivariable linear regression model was fitted to the data to investigate associations between potential prognostic factors (diabetes, smoking, age, BMI) and the mean cumulative drainage fluid over both sides. All secondary endpoints were analyzed in an exploratory manner using two-sided tests on a significance level of 5%. Statistical analysis was performed using IBM SPSS Statistics for Windows, version 25 (IBM Corp., Armonk, N.Y., USA) and R version 3.4.4 (R Foundation for Statistical Computing, Vienna, Austria).

## Results

The trial was conducted from 2013 to 2017 and stopped early after the results of the first planned interim analysis, which was started after the inclusion of 25 patients. During interim analysis, recruiting continued. After evaluation of the interim analysis, it was decided to stop the study early for futility, as there was no significant effect or tendency in favor of study treatment. The study was stopped permanently after 31 included patients. Three patients had previous inguinal surgery while two patients had neoadjuvant systemic therapy for penile cancer. The patient characteristics are shown in Table 2. One patient died in the follow-up phase due to pulmonary embolism while being treated for wound complications in an external hospital. All other patients were alive at the end of the follow-up phase.

**Table 2:** Patient characteristics

Patient characteristics	
Patient number (*n*)	31
Smoker (%)	45
Sentinel-node-dissection (%)	27
Diabetes (%)	10

	Median	Range	IQR
Age (years)	62	34–82	57–70
BMI (kg/m^2^)	28	19–43	24–32

Lymphadenectomy	VAC			CONV		
	Median	Range	IQR	Median	Range	IQR
Number of dissected LN	9	1–20	4–13	7	1–19	6–11
Weight of dissected tissue (g)	52	8–162	39–83	50	5–165	32–81

Tumor stage (final)	pT1		pT2		pT3	
Number of patients (%)	pT1 G1 pN0 cM0	1 (3%)	pT2 pN0 cM0	8 (27%)	pT3 pN0 cM0	2 (7%)
	pT1 G2 pN0 cM0	9 (30%)	pT2 pN1-3 cM0	5 (17%)	pT3 pN1-3 cM0	2 (7%)
	pT1 G3 pN0 cM0	1 (3%)				
	pT1 pN1-3 cM0-1	2 (7%)				

Lymph nodes (pN)	pN0	pN1	pN2	pN3
% of patients	70	13	7	10

Distant metastasis (cM)	cM0 (%)	cM1 (%)
	97	3

### Primary endpoint: cumulative drainage fluid

**Fig. 2:**
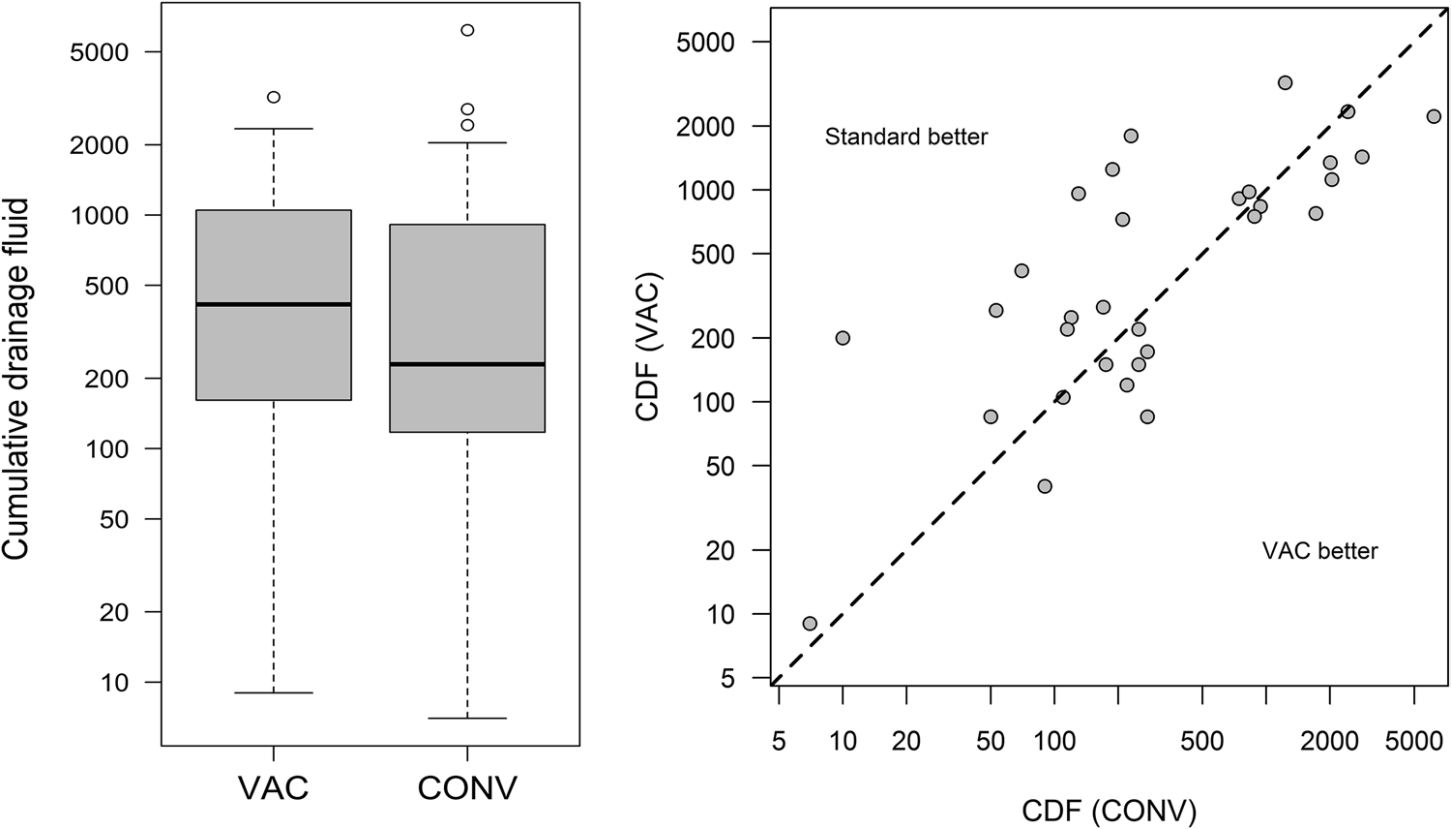
Box plot (left) and scatter plot (right) of cumulative drainage fluid in the VAC vs. CONV group

In 16 patients, the intervention side (VAC) had a smaller CDF compared to the control side (CONV), while in 15 patients the control side had a smaller CDF compared to the intervention side. Consequently, the estimated probability for a smaller CDF by VAC as compared to CONV is 52% (95% confidence interval: 33–70%), which is relevantly smaller than the hypothesized 70% (*p* = 0.031; exact binomial test with *P*_0_ = 70%). Median CDF was 230 ml (IQR: 115–940) for CONV vs. 415 ml (150–1120) for VAC (*p* = 0.673) (Fig. 2).

### Secondary endpoints

**Fig. 3:**
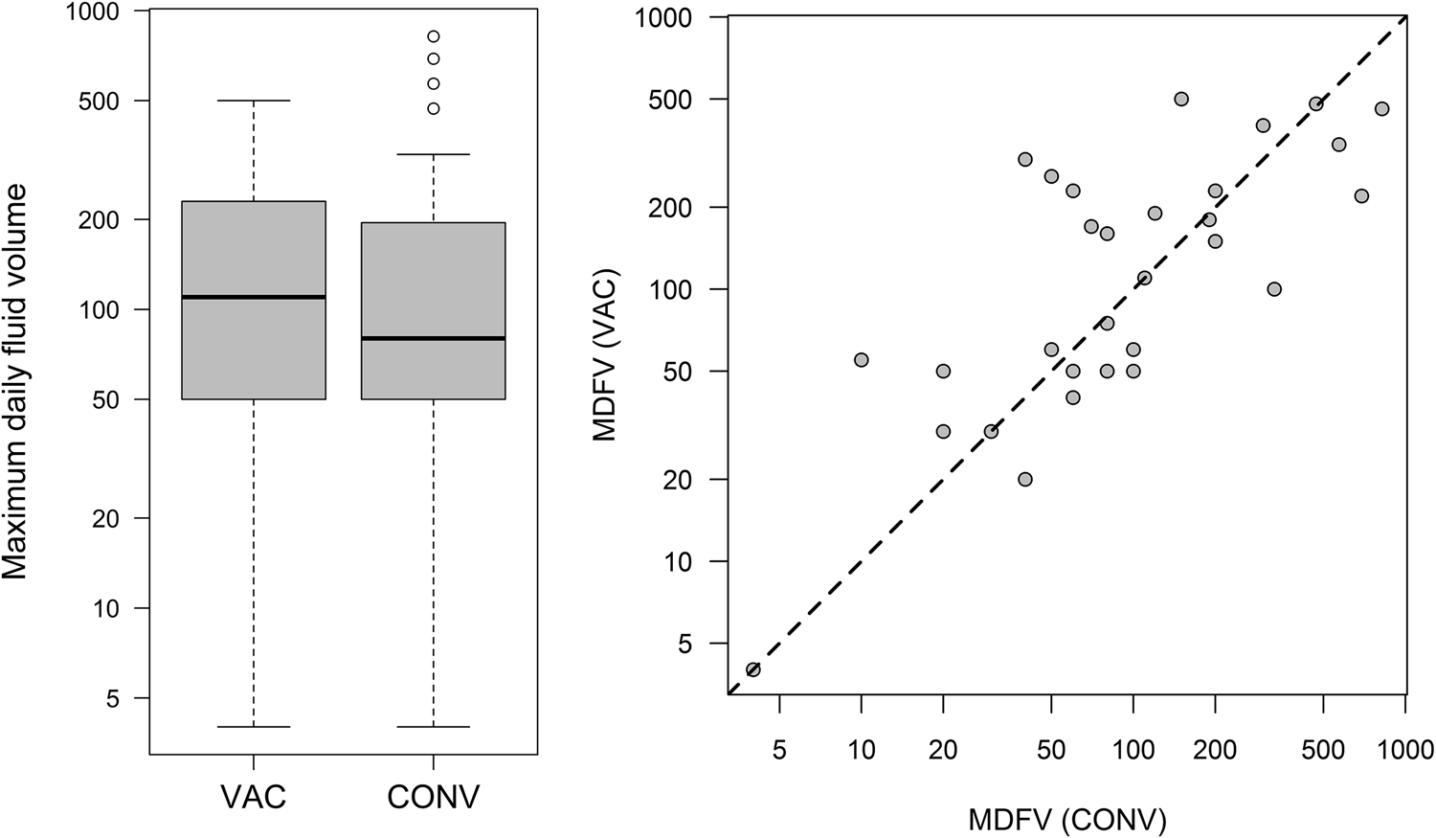
Box plot (left) and scatter plot (right) of maximum daily fluid volume in the VAC vs. CONV group

**Fig. 4:**
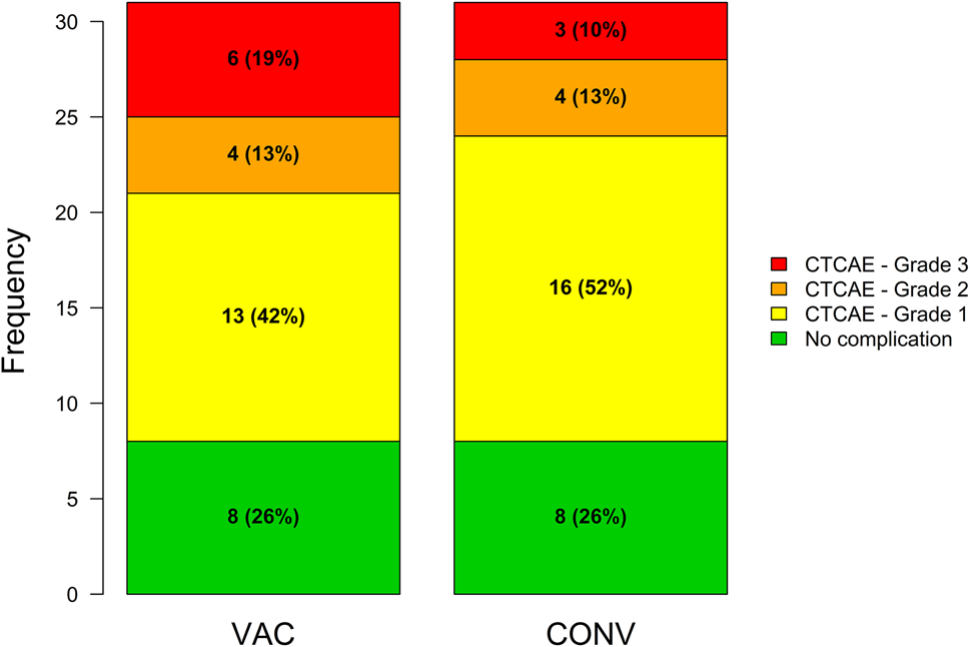
Frequency of complications by grade (CTCAE 4.0) in the VAC vs. CONV group

The median maximum daily fluid volume (MDFV) was 80 ml (IQR: 50–200 ml) for CONV vs. 110 ml (IQR: 30–230 ml) for VAC (Fig. 3). Median time of indwelling drainage was 7 days (5–9 days) for CONV vs. 8 days (5–11 days) for VAC. All grade surgery-related complications were seen in 74% (23/31) patients on the conventional side vs. 74% (23/31) on the VAC side, while grade 3 complications occurred in 3 patients (CONV) vs. 6 patients (VAC) (Table 3; Fig. 4). Prolonged hospital stay due to wound problems occurred in 10 patients (CONV) vs. 15 patients (VAC) (*p* = 0.125) with a median hospital stay of 11.5 days (8–20 days). A reintervention due to complications was needed in 14 patients (45%) receiving CONV and 13 patients (42%) receiving VAC. The median pain and satisfaction levels were 1 (0–3) and 9 (8–10) under CONV vs. 2 (0–2) and 9 (7–10) under VAC (*p* = 0.527 for pain and *p* = 0.255 for satisfaction), showing that both wound treatments had a low pain score and excellent satisfaction level for the patients.

**Table 3:** Postoperative complications

Complication per side/patient	Grade 1			Grade 2			Grade 3		
	VAC	CONV	Overall	VAC	CONV	Overall	VAC	CONV	Overall
Lymphedema (%)	7 (23%)	8 (26%)	9 (29%)	0 (0%)	1 (3%)	1 (3%)	0 (0%)	0 (0%)	0 (0%)
Lymphocele (%)	5 (16%)	10 (33%)	10 (33%)	3 (10%)	2 (6%)	3 (10%)	5 (16%)	3 (10%)	5 (16%)
Lymph leakage (%)	1 (3%)	1 (3%)	1 (3%)	2 (6%)	1 (3%)	3 (10%)	2 (6%)	1 (3%)	3 (10%)
Wound dehiscence/infection (%)	2 (6%)	1 (3%)	2 (6%)	0 (0%)	2 (6%)	2 (6%)	3 (10%)	2 (6%)	3 (10%)
Thromboembolic event (%)	0 (0%)	1 (3%)	1 (3%)	0 (0%)	0 (0%)	0 (0%)	0 (0%)	0 (0%)	0 (0%)

One patient died due to pulmonary embolism in the follow up period

We performed uni- and multivariate analyses investigating the association of potential risk factors (smoking, age, obesity, diabetes) with cumulative drainage fluid, the necessity of reinterventions and occurrence of relevant complications. We did not observe any statistically significant associations, which is likely due to the case number.

## Discussion

We present the results of a prospective, randomized trial to analyze the effect of epidermal vacuum therapy on wound complications after inguinal lymphadenectomy in patients with penile cancer. We did not observe a substantial difference between epidermal vacuum treatment and conventional wound treatment after a planned interim analysis. As the preplanned interim analysis showed that the primary endpoint was highly unlikely to be met after the inclusion of more patients, the study was stopped early for futility.

When designing the study protocol we tried to minimize bias. To exclude patient inherent factors contributing to wound problems, we directly compared both treatments within the same patient, applying the study intervention to one groin and conventional treatment to the other groin. Therefore, if epidermal vacuum therapy would reduce wound problems in reality, it should be evident irrespective of surgical technique, surgeon skill or patient predisposition to complications.

The observed complications and reinterventions are in line with published studies, although more recent publications (including our own retrospective analysis from 2012) have reported fewer complications [16, 17]. In the authors` opinion, this might be in part due to the retrospective design of these reports; showing that we might underestimate complication rates in clinical practice if it is not prospectively and actively assessed. This is especially true as a relevant part of reinterventions was after discharge.

Outside of penile cancer therapy, inguinal incisions are common in vascular surgery to access the femoral vessels. A recent prospective randomized single-center trial evaluated 119 femoral incisions after elective vascular surgery [18]. Wound care was either a gauze dressing or an epidermal vacuum therapy like the one used in our study. They stratified their patients in a low and high-risk cohort. The rate of major wound complications in the high-risk group was 25% in the control group and 8.5% in the epidermal vacuum group (*p* < 0.001), while reoperation/readmission rate was 18.3%/16.7% in the control group vs 8.5%/6.8% in the intervention group, showing a statistically significant benefit for the use of epidermal vacuum dressing in the high-risk group. Another recent prospective randomized trial in vascular surgery found similar results in their analysis [19]. They analyzed 188 patients after vascular surgery of the groin for surgical site infections. Overall, the rate of wound infections was 22.8%. The control group developed more surgical site infections (33.3%) compared to the intervention group (13.2%; *p* = 0.0015). A prospective, controlled trial with a comparable setting to the above-mentioned studies in vascular surgery showed a strong trend in favor of epidermal vacuum dressing but missed statistical significance by a narrow margin [20].

These results seem to contradict the findings in our trial at first glance. But although inguinal lymphadenectomy for penile cancer and femoral vascular surgery share the same anatomical region, they are substantially different in the extent of trauma to lymphatic vessel and removal of lymphatic and fatty tissue. The extended trauma to the lymphatic system could be the reason that the suction, which is generated by an epidermal vacuum system, is just not sufficient to prevent wound complications in this situation. Therefore, these findings are probably not directly comparable.

The results for epidermal vacuum therapy in other anatomical regions are heterogeneous [21], which is probably due to the fact that different regions are under different stress by movements and extent of lymphatic drainage, thus making it difficult to directly compare the efficacy of epidermal vacuum therapy in different indications and anatomical regions.

Limitations of the study are a long recruitment time and a relatively low patient number, which is a general problem in rare diseases like penile cancer. Other limitations, like the use of different surgery techniques (DSNB vs. modified vs. radical lymphadenectomy) or the number or experience of surgeons performing the surgery should not be relevant for the comparison of both treatment arms, due to our study design. On the other hand, these factors may influence the overall complication rate, which was high in both arms. We also reported pain score and satisfaction results per side. Due to the non-blinded application of wound dressing, which was obvious for the patients, these results may be biased.

## Conclusions

There is no obvious solution to reduce the high complication rate of inguinal lymphadenectomy. So far, perfecting surgical technique with meticulous ligation of lymphatic vessels and reducing surgical trauma by using dynamic sentinel node biopsy whenever possible seem to be—in our opinion—the most promising approaches to reduce the morbidity of this intervention.
